# Influence of competitive level and playing position on the physical and technical demands of professional men’s football

**DOI:** 10.5114/biolsport.2026.156233

**Published:** 2026-01-02

**Authors:** Miguel Lampre-Ezquerra, José Luis Arjol-Serrano, Roberto López-Del Campo, Ricardo Resta, Elena Mainer-Pardos, Demetrio Lozano

**Affiliations:** 1 Universidad San Jorge, Autov A23 km 299, Villanueva de Gállego, 50830 Zaragoza, Spain; 2 Department of Competitions and Mediacocach, LaLiga, Torrelaguna 60, 28043 Madrid, Spain

**Keywords:** Soccer, Physical demands, Technical performance, Competitive standards, High-intensity running

## Abstract

This study investigated the differences in physical and technical performance between players in Spain’s First and Second Division professional men’s football leagues, with a specific focus on playing positions and phases of play (in and out of possession). Drawing on data from 1,608 official matches across the 2021/22 and 2022/23 seasons, involving 44 teams, the research employed advanced tracking technologies (TRACAB and Mediacoach) and technical event analysis (OPTA) to provide a comprehensive assessment of physical demands and technical actions. In possession phases, First Division central midfielders (CM) and forwards (FW) covered significantly more distance at high speeds (p = 0.010 and 0.027; ES = -0.49) and during sprints (p = 0.041 and 0.025; ES = -0.45 and -0.59), whereas wide defenders (WD) demonstrated greater high-intensity efforts in both possession and defensive phases (p = 0.002–0.009; ES = -0.50 to -0.59). Conversely, central defenders (CD) and wide midfielders (WM) did not show significant differences in physical demands between divisions. Technically, FW in the First Division recorded higher values in total passes, forward passes, attacking zone passes, successful dribbles, and crosses, while WM showed significantly greater totals in passes, forward passes, and attacking zone passes compared to their counterparts in the Second Division. In contrast, Second Division players, particularly CD and WD, executed longer passes (p = 0.025 and 0.001; ES = 0.29 and 0.20), suggesting a more direct style of play. In conclusion, the study demonstrated that competitive level and playing position significantly influence the physical and technical demands of professional football. The findings suggest that technical proficiency, particularly in offensive play, complements position-specific physical demands in highintensity actions as a key differentiator between divisions.

## INTRODUCTION

From a systemic perspective, football is characterised by a high degree of complexity, wherein a dynamic interaction of cooperation and opposition between two teams generates uncertainty, randomness and a constant need to adapt to the various tactical, collective and individual behaviours that emerge [1] during the four key phases of the game: attacking, defending, defensive-to-offensive transition and offensive-to-defensive transition [2].

The tactical nature of football has a direct impact on physical demands, which are primarily dependent on the specific position of the player [3, 4] and the tactical roles derived from it on the pitch [5–7]. Other contextual factors—such as score line dynamics [8], home versus away status [9], seasonal progression [10, 11], or managerial changes [12]—have also been shown to influence the physical demands placed on football players.

In this regard, the analysis of competitive standard—understood as the level of quality and intensity of play, determined by the competition tier or the relative status of the teams—has received limited and somewhat inconsistent attention in the literature. Early studies revealed that players competing at a higher standard, such as those in Italy’s Serie A, covered 28% and 58% more distance at high intensity and sprinting, respectively (p < 0.05), than sub-elite players in the Danish league [13]. Similarly, Ingebrigtsen et al. observed that players from high-ranked (1^st^–2^nd^) and mid-ranked (5^th^–8^th^) Danish teams sprinted 25% and 33% more (p < 0.001), respectively, than those from lower-ranked teams (10^th^–12^th^) [14].

However, these studies reported high-intensity (> 18 km/h) and sprinting distances (> 30 km/h) which are considerably higher than those typically observed in contemporary football. These findings contradict more recent scientific evidence indicating that physical demands have increased over successive seasons [10, 11], suggesting that the validity of earlier benchmarks may be questionable. Methodological issues (e.g., definition of speed thresholds, analytical methods employed to quantify high-speed distances) and the inclusion of players from different European leagues with distinct competitive standards may partly explain the differences across studies. Nevertheless, Gómez Piqueras et al. reported similar findings in a more recent study conducted within the Spanish leagues [15]. Players from the first division teams covered significantly greater distances at high intensity (21–24 km/h) and sprinting speeds (> 24 km/h) (p < 0.01; ES = 1.2) than those from the second division teams, although no differences were observed in the total distance covered [15]. In contrast Bradley et al., in a comparative study of players from the three main professional leagues in England, found that players in League One (third tier) and the Championship (second tier) covered greater total and high-intensity distances (≥ 19.8 km/h) (20–34%, p < 0.01) than those in the Premier League (first tier) across all playing positions [16]. Similarly, Di Salvo et al. reported a 7% difference in high-intensity running distance (≥ 19.8 km/h) between Championship and Premier League players [17].

Within the context of Spanish professional football, and in line with these findings, Lago-Peñas et al. observed that when effective playing time was considered, second division teams (LaLiga Smartbank) covered greater total distances (p < 0.01) than first division teams (LaLiga Santander) [18]. However, when the total match time was used as the reference, the first division teams recorded greater sprint distances (≥ 21 km/h) (p < 0.01) than their second division counterparts.

Despite these findings, although several studies have investigated the differences between playing positions [6, 19, 20], none to date have examined how these differences manifest across competitive standards. In this regard, it has been demonstrated that CM tend to cover the greatest total distances during matches [3, 19]. With respect to high-intensity running, wide defenders (WD) and wide midfielders (WM) cover more distance than central defenders (CD) and forwards (FW). Wide midfielders cover the greatest sprint distances during matches [19].

In recent years, aspects related to physical performance have been extensively examined in the scientific literature. However, the key determinants of performance and affiliation with a higher competitive standard in football may be more closely associated with technical-tactical indicators and collective behaviours than with conditional performance alone [17, 21]. Therefore, studying these elements in conjunction with physical aspects is essential for a more comprehensive analysis [5].

Metrics such as the number of completed passes, frequency of forward passes, total passes, and average touches per possession were approximately 5%–40% higher in the Premier League than in lower-tier competitions [17]. These findings likely indicate that players competing at higher levels are more technically and tactically efficient, and that the playing model at this competitive standard is far more possession-oriented than direct play—an approach that directly influences physical demands. Nevertheless, as many studies do not differentiate players by playing position, these results are relatively general. This is a critical limitation, given that each position requires distinct physical and technical skills, with specific roles and responsibilities within the match context [5–7].

To the best of our knowledge, no study has conducted a comparative analysis of physical and technical aspects by playing position across the different phases of play—with and without ball possession—between the two main tiers of professional men’s football in Spain. Therefore, this study aimed to identify the differences in physical and technical performance, both in possession and out of possession, for each playing position between teams in the Spanish first and second divisions. We hypothesise that technical aspects and successful actions of WD, CM, and WM during the possession phase may exert a greater influence than physical demands in determining the affiliation with a higher competitive standard.

## MATERIALS AND METHODS

### Experimental design

This study was descriptive and employed an observational methodology, applied through data obtained via electronic tracking and performance monitoring systems. The aim was to analyse and compare the physical demands and technical actions experienced by professional football players across two different competitions (Spain’s First and Second Divisions) over the course of two seasons (2021/22 and 2022/23), encompassing a total of 1,608 official matches. Positional consistency was further verified by two UEFA-qualified coaches who observed each match [22], and their assessments were complemented by Mediacoach player heat maps. Data for players who did not play the entire match were excluded for further analysis.

### Participants

The sample was collected from 1,608 individual match observations involving 1,050 male professional football players from 44 teams competing in Spain’s First and Second Divisions. All players trained for approximately 10 hours per week and participated in one official match during the weekend. Occasionally, teams played an additional midweek match in European competitions or the Copa del Rey. Players were grouped according to their playing positions, defined as their location within the team’s tactical formation. The selected positions included CD, WD, CM, WM, and FW. Goalkeepers were excluded from the analysis. The data were obtained through match monitoring systems, and researchers were authorised to use the collected data; therefore, no ethics committee approval was required [23]. Furthermore, this study was conducted in accordance with the Declaration of Helsinki (2013) and the ethical standards of research in sport and exercise science [24], as well as the regulations of the Spanish Professional Football League (LaLiga). No information that could identify individual players was included in the analysis (LaLiga General Assembly, 2019) [25].

### Physical demands

The physical performance profiles of the players were monitored during each match using TRACAB, a multi-camera computerised optical tracking system (ChyronHego VID, New York, NY) operating at a sampling frequency of 25 Hz, in conjunction with the Mediacoach software (LaLiga, Madrid, Spain). All running performance data analysed in this study were obtained exclusively from this system. The reliability of this tracking system has been evaluated in previous studies [26–28]. However, caution is still advised when interpreting accelerations and decelerations greater than 3 m/s^2^ recorded during matches using this system [29]. Mediacoach tends to register higher values than GPS devices for high-intensity accelerations and decelerations (> 3 m/s^2^), likely due to differences in how each system defines the acceleration onset [29]. This discrepancy, also reported by other authors [30], led us to exclude such data from the present analysis to avoid potentially misleading conclusions.

### Technical actions

The events were obtained via OPTA (Opta Sports, Spain) using the Mediacoach software (LaLiga, Madrid, Spain). In previous studies, the reliability of OPTA (Spain) has shown intraclass correlation coefficient values ranging from 0.88 to 1.00 [27]. The technical actions **Table 1**. Technical performance variables and definitions have been previously analysed [9, 31, 32], and the associated definitions are presented in Table 1.

**TABLE 1:** Technical performance variables and definitions

Technical variables in possession	
Total pass (Nº)	Total number of passes
Total pass success (%)	Percentage of total number of accurate passes
Long pass (Nº)	Total number of long passes at a distance equal to or greater than 30 m
Long pass success (%)	Percentage of total number of accurate long passes at a distance equal to or greater than 30 m
Forward pass (Nº)	Total number of passes heading forward
Forward pass success (%)	Percentage of total number of accurate passes heading forward
Attack zone pass (Nº)	Total number of passes in the last third of the field
Goal shot (Nº)	Total number of shots to score
Goal shot on target (Nº)	Number of shots within a goal
Crosses (Nº)	Total number of crosses
Crosses success (%)	Percentage of total number of accurate crosses
Dribbles (Nº)	Total number of overflow actions of an attacker on a defender
Turnover (Nº)	Number of times a team loses possession of the ball during the game

**Technical variables out of possession**	

Recovery (Nº)	Number of ball recoveries made by a team that result in a change in possession of the ball between teams.
Opposite field recovery (Nº)	Number of ball recoveries made by a team that result in a change in possession of the ball between teams in the opposite field

### Statistical analysis

SPSS for Windows, version 25.0 (IBM Corp., Armonk, NY, USA) was used for statistical analysis. Statistical normality and homogeneity of variances were assessed using the Kolmogorov–Smirnov and Levene’s tests, respectively. Parametric techniques were applied when the analysed variables followed a normal distribution, whereas nonparametric tests were used when the variables did not meet this assumption. Out of the 130 variables analysed, 76 met the normality assumptions and were tested using parametric methods, whereas 7 variables did not meet these assumptions and were analysed with non-parametric methods. Physical demands and technical–tactical indicators were analysed using the independent samples t-test (parametric technique) and the Mann–Whitney U test (non-parametric technique) to compare differences in playing position, ball possession, and non-possession phases between First and Second Division football players. Results are presented as mean ± standard deviation (± SD). Statistical significance was set at *p* < 0.05. Additionally, the magnitude of between-group differences was expressed as the standardised mean difference (Cohen’s effect size [ES], with 90% confidence intervals [CI]). The criteria for interpreting the magnitude of the ES were: < 0.2 trivial, > 0.2 to 0.6 small, > 0.6 to 1.2 moderate, and > 1.2 large [33].

## RESULTS

Table 2 describes the physical demands of First and Second Division football players by playing position and across different phases of play.

**TABLE 2:** Description of the physical demands (mean ± SD) of football players for each playing position in the first and second divisions

	CD		WD		CM		WM		FW	

	1^st^ division	2^nd^ division	1^st^ division	2^nd^ division	1^st^ division	2^nd^ division	1^st^ division	2^nd^ division	1^st^ division	2^nd^ division
TD (m)	9655.8 ± 293.7	9677.3 ± 337.2	10302.4 ± 360.8	10444.8 ± 300.3	11172.0 ± 308.1	11035.3 ± 380.9	10548.3 ± 414.9	10584.4 ± 569.5	10204.4 ± 426.7	10112.3 ± 451.0

TD-A (m)	3335.6 ± 437.4	3287.8 ± 354.3	3464.5 ± 512.7	3660.8 ± 422.2	4014.7 ± 524.0	3890.7 ± 507.4	3956.7 ± 415.7	3940.7 ± 374.0	3968.6 ± 442.1	3850.9 ± 371.5

TD-D (m)	3915.3 ± 309.0	3858.1 ± 296.7	4080.3 ± 358.3	4012.6 ± 264.3	4585.3 ± 380.3	4472.3 ± 343.9	3855.0 ± 429.5	3850.6 ± 415.8	3649.5 ± 416.2	3586.0 ± 319.2

HSR (m)	426.3 ± 71.3	408.0 ± 53.8	737.9 ± 87.1	721.7 ± 94.2	525.1 ± 85.1	485.7 ± 82.1	797.5 ± 125.8	755.0 ± 116.9	676.6 ± 111.1	631.8 ± 130.7

HSR-A (m)	78.8 ± 34.6	77.6 ± 18.7	316.4 ± 62.6	322.8 ± 79.5	165.3 ± 47.2	145. 0 ± 55.2	± 54.5	± 77.4	± 71.5	± 88.9

HSR-D (m)	337.1 ± 49.2	316.5 ± 46.7	411.8 ± 47.9	386.6 ± 37.5	352.2 ± 62.8	332.4 ± 41.2		311.3 ± 64.3	242. 7 ± 59.0	237.5 ± 54.6

SPR (m)	206.8 ± 42.9	195.0 ± 31.2	393.8 ± 64.1	386.7 ± 61.1	224.6 ± 48.9	206.8 ± 46.5	426.0 ± 92.6	394.2 ± 79.2	351.3 ± 73.7	317.6 ± 86.0

SPR-A (m)	32.2 ± 18.5	30.7 ± 9.9	181.5 ± 41.3	186.2 ± 51.0	72.1 ± 30.1	61.3 ± 29.6	267.7 ± 80.4	238.2 ± 56.9	232.7 ± 52.1	204.1 ± 60.7

SPR-D (m)	171.7 ± 30.4	160.1 ± 27.6	213.9 ± 28.3	196.6 ± 22.7	150.3 ± 32.3	143.3 ± 23.9	154.8 ± 35.7	152.2 ± 38.8	115.7 ± 32.9	110.6 ± 33.3

NºSPR (n)	11.7 ± 2.2	11.1 ± 1.7	21.0 ± 2.7	20.2 ± 2.8	12.4 ± 2.5	11.7 ± 2.3	22.3 ± 4.1	20.9 ± 3.9	19.3 ± 3.8	17.8 ± 4.3

Vmax (km · h)	30.7 ± 0.7	30.6 ± 0.5	31.8 ± 0.5	31.6 ± 0.5	29.8 ± 0.6	29.9 ± 0.6	31.9 ± 0.9	31.8 ± 1.0	31.9 ± 0.8	31.2 ± 1.1

*Note*: CD: central defenders; WD: wide defenders; CM: central midfielders; WM: wide midfielders; FW: forwards; TD: total distance; TD-A: total distance with possession; TD-D: total distance without possession; HSR: distance covered above 21.0 km · h^−1^; HSR-A: distance covered with possession above 21.0 km · h^−1^; HSR-D: distance covered without possession above 21.0 km · h^−1^; SPR: distance covered above 24.0 km · h^−1^; SPR-A: distance covered with possession above 24.0 km · h^−1^; SPR-D: distance covered without possession above 24.0 km · h^−1^; NºSPR: number of sprints above 24.0 km · h^−1^; Vmax: maximal speed.

Table 3 shows that First Division WD exhibited significantly greater demands (*p* = 0.009–0.002) in distance covered without possession above 21.0 km · h^−1^ (HSR-D) and distance covered with possession above 21.0 km · h^−1^ (HSR-A), with small-to-moderate effect sizes (ES = -0.50 and -0.59). First Division CM also covered greater distances in HSR, HSR-A, and distance covered with possession above 24.0 km · h^−1^ (SPR-A) (*p* = 0.010–0.041; ES = -0.45 to -0.49, small-to-moderate), as did FW, who displayed higher values in HSR-A, and SPR-A (*p* = 0.027–0.025; ES = -0.49 and -0.50, small-to-moderate) compared to Second Division players.

**TABLE 3:** Mean differences (MD, %) and effect sizes (ES; ± CL) of the physical demands (mean ± SD) encountered by football players for each playing position between the first and second divisions

	CD		WD		CM		WM		FW	

	MD (%)	ES	MD (%)	ES	MD (%)	ES	MD (%)	ES	MD (%)	ES
TD	0.4	0.13; ± 0.36	1.6	0.50; ± 0.37	-1.2	-0.38; ± 0.36	0.4	0.08; ± 0.37	-1.0	-0.22; ± 0.37
TD-A	-1.1	-0.09; ± 0.37	0.7	0.05; ± 0.37	-3.1	-0.24; ± 0.36	-0.4	-0.03; ± 0.38	-2.9	-0.27; ± 0.27
TD-D	-1.0	-0.13; ± 0.37	-1.1	-0.15; ± 0.37	-2.2	-0.27; ± 0.37	0.3	0.03; ± 0.38	-1.6	0.14; ± 0.37
HSR	-3.3	-0.22; ± 0.37	-2.0	-0.16; ± 0.36	-7.3	-0.46; ± 0.37*	-4.5	-0.27; ± 0.38	-6.9	-0.36; ± 0.36
HSR-A	4.2	0.12; ± 0.37	1.2	0.05; ± 0.36	-14.4	-0.49; ± 0.36*	-4.7	-0.18; ± 0.38	-10.1	-0.49; ± 0.36*
HSR-D	-5.5	-0.28; ± 0.50	-5.4	-0.50; ± 0.36**	-4.7	-0.32; ± 0.37	-2.0	-0.10; ± 0.38	-1.3	-0.05; ± 0.37
SPR	-4.5	-0.24; ± 0.33	-1.5	-0.09; ± 0.37	-8.2	-0.38; ± 0.37	-6.1	-0.26; ± 0.38	-10.5	-0.42; ± 0.37
SPR-A	4.4	0.09; ± 0.37	1.3	0.05; ± 0.37	-18.0	-0.45; ± 0.37*	-7.5	-0.23; ± 0.38	-13.7	-0.50; ± 0.36*
SPR-D	-6.1	-0.35; ± 0.37	-7.6	-0.59; ± 0.37**	-4.1	-0.21; ± 0.37	-1.2	-0.05; ± 0.38	-4.3	-0.13; ± 0.37
NºSPR	-3.7	-0.21; ± 0.37	-3.6	-0.27; ± 0.36	-5.9	-0.30; ± 0.37	-5.2	-0.26; ± 0.38	-8.1	-0.35; ± 0.36
VMax	-0.3	-0.16; ± 0.37	-0.6	-0.37; ± 0.37	0.2	0.12; ± 0.36	-0.1	-0.04; ± 0.36	-0.4	-0.13; ± 0.36

*Note*: CD: central defenders; WD: wide defenders; CM: central midfielders; WM: wide midfielders; FW: forwards; TD: total distance; TD-A: total distance with possession; TD-D: total distance without possession; HSR: distance covered above 21.0 km · h^−1^; HSR-A: distance covered with possession above 21.0 km · h^−1^; HSR-D: distance covered without possession above 21.0 km · h^−1^; SPR: distance covered above 24.0 km · h^−1^; SPR-A: distance covered with possession above 24.0 km · h^−1^; SPR-D: distance covered without possession above 24.0 km · h^−1^; NºSPR: Number of sprints above 24.0 km · h^−1^; Vmax: maximal speed.

* Significant at p < 0.05;

** Significant at p < 0.01.

Table 4 presents the offensive and defensive technical actions of First and Second division football players, according to their playing positions.

**TABLE 4:** Description of the technical demands (mean ± SD) encountered by football players for each playing position in the first and second divisions

	CD		WD		CM		WM		FW	

	1^st^ division	2^nd^ division	1^st^ division	2^nd^ division	1^st^ division	2^nd^ division	1^st^ division	2^nd^ division	1^st^ division	2^nd^ division
TP (Nº)	47.35 ± 11.30	46.82 ± 12.16	49.36 ± 11.03	47.14 ± 7.53	47.54 ± 13.21	44.44 ± 10.59	35.19 ± 8.84	31.71 ± 9.99	22.85 ± 7.15	18.47 ± 4.75

TPS (%)	39.66 ± 11.67	38.44 ± 11.99	37.85 ± 10.96	34.06 ± 7.03	39.42 ± 12.90	35.77 ± 10.11	26.31 ± 8.02	22.72 ± 8.33	15.9 ± 6.03	12.11 ± 3.55

LP (Nº)	7.09 ± 1.94	7.66 ± 2.04	7.17 ± 2.29	8.66 ± 2.42	5.04 ± 2.39	5.45 ± 2.31	3.34 ± 1.85	3.59 ± 2.53	1.43 ± 1.15	0.90 ± 0.62

LPS (%)	3.43 ± 1.05	3.53 ± 1.24	3.40 ± 1.07	3.84 ± 1.15	2.83 ± 1.57	2.93 ± 1.37	1.60 ± 0.97	1.67 ± 1.29	0.74 ± 0.56	0.43 ± 0.31

FP (Nº)	35.0 ± 8.07	34.6 ± 8.7	30.33 ± 6.66	30.03 ± 4.98	29.56 ± 8.94	27.88 ± 7.08	18.35 ± 5.87	17.52 ± 7.07	11.22 ± 4.35	9.30 ± 3.03

FPS (%)	27.55 ± 8.47	26.45 ± 8.59	20.07 ± 6.20	18.18 ± 4.08	22.47 ± 8.63	20.23 ± 6.32	11.55 ± 4.77	10.39 ± 5.23	6.18 ± 3.05	4.68 ± 1.79

AZP (Nº)	1.75 ± 1.94	1.30 ± 1.40	8.20 ± 3.93	6.90 ± 2.36	8.89 ± 4.77	6.69 ± 3.37	12.62 ± 4.44	10.45 ± 2.99	9.08 ± 3.52	7.59 ± 2.09

GS (Nº)	0.48 ± 0.25	0.45 ± 0.23	0.54 ± 0.31	0.56 ± 0.34	0.99 ± 0.47	0.92 ± 0.53	1.84 ± 0.80	1.74 ± 0.70	2.43 ± 0.73	2.19 ± 0.58

GST (Nº)	0.13 ± 0.11	0.13 ± 0.10	0.14 ± 0.14	0.15 ± 0.12	0.32 ± 0.22	0.27 ± 0.23	0.70 ± 0.37	0.62 ± 0.31	0.98 ± 0.39	0.90 ± 0.32

CR (Nº)	0.29 ± 0.56	0.23 ± 0.57	3.05 ± 1.45	3.53 ± 1.62	1.45 ± 1.52	1.37 ± 1.54	3.36 ± 2.15	2.97 ± 2.01	1.04 ± 1.38	0.60 ± 0.57

CRS (%)	0.06 ± 0.14	0.05 ± 0.15	0.68 ± 0.39	0.82 ± 0.52	0.39 ± 0.48	0.37 ± 0.49	0.79 ± 0.60	0.68 ± 0.61	0.22 ± 0.36	0.10 ± 0.12

DR (Nº)	0.35 ± 0.35	0.33 ± 0.39	1.57 ± 0.83	1.61 ± 0.84	1.43 ± 0.90	1.36 ± 0.92	3.81 ± 1.88	3.48 ± 1.62	2.12 ± 1.13	1.91 ± 0.93

TO (Nº)	5.70 ± 1.46	6.09 ± 1.08	5.57 ± 0.95	5.51 ± 0.97	5.58 ± 1.28	5.92 ± 1.36	3.82 ± 1.01	3.84 ± 1.01	2.20 ± 0.85	2.10 ± 0.80

RC (Nº)	0.71 ± 0.3	0.63 ± 0.3	1.12 ± 0.37	1.05 ± 18.5	1.76 ± 0.52	1.82 ± 0.5	1.35 ± 0.42	1.39 ± 0.38	1.06 ± 0.36	1.05 ± 0.36

RCOF (Nº)	3.74 ± 1.08	3.90 ± 1.02	6.62 ± 1.33	7.05 ± 1.04	6.13 ± 1.60	6.15 ± 1.61	8.43 ± 1.61	8.78 ± 1.71	9.05 ± 1.84	9.19 ± 1.87

*Note*: TP: total pass; TPS: total pass success; LP: long pass; LPS: long pass success; FP: forward pass; FPS: forward pass success; AZP: attack zone pass; GS: goal shot; GST: goal shot on target; CR: crosses; CRS: crosses success; DR: dribbles; TO: turnover; RC: recoveries; RCOF: recoveries opposite field.

*Significant at p < 0.05;

**Significant at p < 0.01.

Table 5 shows that the First Division CD performed significantly more attacking zone passes (AZP) and recoveries in the opponent’s half (RCOF) (*p* = 0.026–0.005), with small-to-trivial effect sizes (ES = -0.26 and -0.19). However, in the second division, CD executed longer passes (LP) (*p* = 0.025; ES = 0.29, small). First Division WD display higher values in total passes (TPS), forward passes (FPS), and AZP (*p* = 0.008–0.026; ES = -0.30 to -0.37, small), whereas WD in the Second Division displayed higher values in LP, long pass success (LPS), crosses (CR), and turnovers (TO) (*p* = 0.001–0.020; ES = 0.25 to 0.64, small to moderate). Regarding CM, those in the First Division showed higher values in TPS, FPS, AZP, successful dribbles (GS), and total dribbles (GST) (*p* = 0.001–0.042; ES = -0.20 to -0.47, trivial to small-to-moderate), while Second Division CM perform more recoveries (RC) (*p* = 0.009; ES = 0.24, small). Finally, WM and FW in the First Division exhibited greater offensive demands, with significantly higher values in indicators such as TP, TPS, FP, FPS, and AZP for WM (*p* = 0.001–0.048; ES = -0.21 to -0.52, trivial to moderate), and in TP, TPS, LP, LPS, FP, FPS, AZP, GS, CR, and successful crosses (CRS) for FW (*p* = 0.001–0.016; ES = -0.29 to -0.82, small to moderate), compared to Second Division players.

**TABLE 5:** Mean differences (MD, %) and effect sizes (ES; ± CL) of the technical demands (mean ± SD) encountered by football players for each playing position between first and second division

	CD		WD		CM		WM		FW	

	MD (%)	ES	MD (%)	ES	MD (%)	ES	MD (%)	ES	MD (%)	ES
TP (Nº)	-1.5	-0.06; ± 0.20	-3.7	-0.20; ± 0.21	-6.0	-0.24; ± 0.17	-11.7	-0.44; ± 0.20**	-18.2	-0.72; ± 0.21**
TPS (%)	-3.4	-0.11; ± 0.20	-8.6	-0.37; ± 0.20**	-8.7	-0.30; ± 0.17*	-15.5	-0.51; ± 0.19**	-22.7	-0.82; ± 0.21**
LP (Nº)	8.4	0.29; ± 0.19*	22.2	0.64; ± 0.20**	10.5	0.21; ± 0.17	-0.1	0.00; ± 0.20	-32.3	-0.51; ± 0.21**
LPS (%)	1.7	0.05; ± 0.19	13.1	0.39; ± 0.20**	2.9	0.05; ± 0.17	-6.9	-0.10; ± 0.20	-38.2	-0.68; ± 0.22**
FP (Nº)	-1.5	-0.06; ± 0.20	0.0	0.00; ± 0.21	-5.1	-0.18; ± 0.17	-7.0	-0.21; ± 0.20*	-15.5	-0.48; ± 0.21**
FPS (%)	-4.3	-0.14; ± 0.20	-7.6	-0.30; ± 0.21**	-8.9	-0.27; ± 0.37*	-13.0	-0.34; ± 0.19**	-21.8	-0.63; ± 0.21**
AZP (Nº)	-17.5	-0.26; ± 0.20*	-12.7	-0.30; ± 0.21*	-23.7	-0.47; ± 0.17**	-15.8	-0.52; ± 0.20**	-14.4	-0.49; ± 0.21**
GS (Nº)	-4.0	-0.07; ± 0.19	3.8	0.06; ± 0.21	-10.6	-0.20 ± 0.17*	-5.0	-0.11; ± 0.20	-8.3	-0.29; ± 0.21**
GST (Nº)	-4.6	-0.10; ± 0.22	1.9	0.04; ± 0.24	-12.6	-0.20; ± 0.18*	-16.5	-0.32; ± 0.20	-5.0	-0.13; ± 0.21
CR (Nº)	-18.2	-0.22; ± 0.24	16.3	0.25; ± 0.21*	-11.5	-0.10; ± 0.18	-12.1	-0.17; ± 0.20	-27.8	-0.35; ± 0.21*
CRS (%)	-9.7	-0.16; ± 0.37	19.3	0.29; ± 0.20	-6.2	-0.06; ± 0.20	-14.6	-0.19; ± 0.20	-25.8	-0.44; ± 0.24**
DR (Nº)	-1.5	-0.02; ± 0.20	1.9	0.03; ± 0.20	-9.2	-0.13; ± 0.17	-7.6	-0.16; ± 0.19	-9.0	-0.17; ± 0.21
TO (Nº)	4.9	0.18; ± 0.16	7.5	0.40; ± 0.21**	0.2	0.01; ± 0.17	3.9	0.19; ± 0.20	1.2	0.06; ± 0.19
RC (Nº)	16.4	-0.31; ± 0.19	-1.1	-0.06; ± 0.21	6.0	0.24; ± 0.31**	0.6	0.02; ± 0.19	-4.5	-0.12; ± 0.21
RCOF (Nº)	-9.5	-0.19; ± 0.19**	-4.9	-0.15; ± 0.19	4.1	0.12; ± 0.17	4.0	0.12; ± 0.19	-1.2	-0.03; ± 0.21

*Note*: TP: total pass; TPS: total pass success; LP: long pass; LPS: long pass success; FP: forward pass; FPS: forward pass success; AZP: attack zone pass; GS: goal shot; GST: goal shot on target; CR: crosses; CRS: crosses success; DR: dribbles; TO: turnover; RC: recoveries; RCOF: recoveries opposite field.

* Significant at p < 0.05;

** Significant at p < 0.01.

## DISCUSSION

The aim of this study was to analyse the physical and technical performance of teams competing in the Spanish First and Second Division football leagues by playing position during phases of ball possession and non-possession to determine whether differences exist between the two main competitions in professional men’s football in Spain. In general, (i) First Division players covered greater HSR and SPR, particularly CM and FW, showing higher values during ball possession, while WD exhibited elevated HSR across both possession and out-ofpossession phases. (ii) With respect to technical performance, First Division players demonstrated superior passing, greater offensive contributions, and more successful attacking actions across most positions, whereas Second Division players executed more defensive actions for CD and CM, as well as certain technical offensive actions, such long passes for CD and WD. These results underscore the combined influence of physical and technical performance by playing position on the competitive standard of the players. This study provides one of the most comprehensive analyses to date on physical and technical performance by playing position in professional football, based on more than 1,600 official matches across two full seasons of the Spanish First and Second Divisions. Overall, our findings are in line with previous research, showing that First Division players covered greater distances at high intensity (21–24 km/h) and sprinting speed (> 24 km/h) than Second Division players [15, 18, 25, 34]. In contrast to our findings, Bradley et al. did not observe differences in the conditional demands placed on football players across three competitive standards within the English League. These discrepancies may be explained by comparing two distinct competitive contexts, each characterised by a different style of play. In the Spanish LaLiga, teams tend to adopt a possession-based style of play [35], which directly influences conditional demands [36]. The present study provides novel insights by conducting a more detailed analysis based on playing positions and phases of the game, which has enabled the identification of when (attacking or defensive phase) and where (playing position) these differences between competitive standards occur.

First, more HSR-D and SPR-D, as well as greater demands in offensive technical aspects (TPS, FPS, and AZP), associated with a more possession-based style of play, were found in WD in the First Division. In contrast, WD in the Second Division performed actions such as LP, LPS, and TO, which are typically linked to a more direct style of play. Wide defenders should actively participate in both the attacking and defensive phases in modern football [11]. Therefore, these physical differences between divisions could be explained by the dual role of performing numerous offensive actions during ball possession [5, 6] and transition into the defensive phase to make the team compact and limit the opponent’s space during defensive phases of play [37]. This defensive aspect demands greater effort from First Division WD due to their prior involvement in attacking actions. In line with this, Yi et al. found that teams characterised by a more possession-based style of play—such as those in which First Division WD operate—covered greater distances at sprinting and high-intensity speeds than teams employing a more direct style of play (ES: 0.33–0.47). Therefore, this disparity in playing style and the use of long ball tactics typically applied in lower competitive standards likely affect the conditional development of WD, which may help explain our findings. This is consistent with previous research showing that lower-ranked or lower-division teams tend to adopt more direct strategies, particularly when playing in defensive contexts or under disadvantageous conditions [15, 38]. Furthermore, the physical performance of WD is also shaped by the tactical system. Previous studies have shown that WD cover greater total and high-intensity distances in three-defender formations [39, 40]. Although the four-defender system is more prevalent in the Spanish First Division, previous studies have not specified the phase of play in which these greater demands for high-intensity actions occur. This may help explain our findings, as WD are required to cover more distance during the defensive phase in a four-defender system, whereas in three-defender formations they receive greater support in fulfilling that role.

Secondly, CM in the First Division showed higher values in HSR, HSR-A, and SPR-A, as well as superior technical performance indicators, such as TPS, FPS, AZP, GS, and GST, compared to CM in the Second Division. Only the defensive technical variable, RC, was higher in the Second Division for CM. These findings align with our results and may explain why First Division CM cover greater distances at high intensity and sprinting speeds during possession phases than those at lower levels. Ju et al. reported that, despite covering similar HSR distances, high-ranking CM (1^st^–5^th^) covered between 78% and 112% more high-intensity distance in attacking actions such as “run with ball” and “break into a box” than lower-ranking CM (6^th^–15^th^) (p < 0.01) [31]. Furthermore, Bradley et al. found that Premier League players performed a significantly greater number (p < 0.01) of total passes, successful passes, forward passes, and touches per possession compared to players in the Championship and League One (ES: 0.3–0.6) [16]. The more frequent execution of these contextualised technical and physical actions in attack by First Division CM appears to reflect a greater responsibility in offensive aspects of play compared to their Second Division counterparts. This includes both the build-up phase, aimed at maintaining possession through more TPS, FPS, and AZP, and the final third, where more HSR-A, SPR-A, GS, and GST are required to exploit space and create goal-scoring opportunities. In contrast, CM in the Second Division may be more associated with a defensive role, involving fewer technical interventions and less attacking movement, but with a greater focus on defensive duties, as evidenced by their higher number of RC in our results.

Third, greater demands in HSR-A and SPR-A, as well as in all technical variables related to offensive play, were required of FW in the First Division than those in the Second Division. These findings indicate that one of the main differences between divisions for FW lies in their active involvement in attacking play, as evidenced by a higher number of technical actions. Forwards must be more dynamic; therefore, their conditional demands appear to be higher during the attacking phase. It is well known that sprinting is the most frequent action in goal-scoring situations, both for the scorer and the assisting player [41], which may be more necessary to create goalscoring opportunities and increase the chances of winning matches in the First Division than in the Second Division. Similar results have been previously reported, showing that players competing at higher standards exhibit higher levels of technical performance [31]. Moreover, teams characterised by a more possession-based style of play— such as those identified in Spanish First Division football—tend to have players, particularly FW and WM, with higher values in all variables related to goal-scoring, attacking play and passing [19, 36], as reflected in the performance of FW in the First Division compared to those in the Second Division.

Lastly, CD and WM did not show differences in physical performance variables, despite exhibiting different technical demands between competitions. In line with this, CD did not show differences in physical demands when winning or losing, or when playing at home or away [3], suggesting that physical performance in CD may not be a key indicator of performance or of belonging to a higher competitive standard.

Greater TP, TPS, FP, FPS, and AZP were performed by WM in the First Division. These results indicate that WM at a higher competitive standard are more technically and tactically competent, and that the style of play at this level may also reflect a game model that is much more possession-oriented. However, this does not appear to affect their physical demands. One possible explanation for these findings is that WM cover most of their high-intensity attack distance by running along the flanks to deliver crosses [5]. In our observations, no differences were found in this indicator, which may indicate a similar number of high-intensity efforts to deliver crosses across divisions. Furthermore, the wide variability in player profiles occupying this position—such as WM playing on their natural foot, with more linear runs and less involvement in build-up play; WM playing on the opposite foot, with greater associative play; or CM deployed as WM to enhance collective defensive performance—could influence the results obtained. In contrast to our findings, greater highintensity distance in the defensive phase while performing ‘covering’ was found in lower-ranked WM (16^th^–20^th^) compared to higherranked WM (1^st^–5^th^) (p < 0.05) [31]. It appears that WM may have greater defensive responsibilities when playing against stronger teams [42], which could explain these differences in relation to our findings, as the comparison in that study was not between competitive standards, but rather among WM within the same league.

The main limitation of this study was that tactical formations and playing styles used in each match were not considered. Future research should account for these factors, as well as coaching changes throughout the season, which are often accompanied by changes in playing models and systems. Another limitation is that the effective playing time was not explicitly considered. Previous research in LaLiga has shown that most physical performance differences between the First and Second Divisions tend to diminish or even disappear when effective playing time is taken into account [18]. However, our inclusion of play phases (possession and non-possession) already provides an effective-context framework. The integration of time-based effective metrics could be a valuable approach for future research, as it would provide a more accurate comparison between competitive levels.

### Practical applications

These results have three clear implications for training. First, competitive standard and playing position influence the physical demands and technical profile of football players. Second, a key practical implication for coaches is to understand the role of superior technical performance at higher competitive levels during the attacking phase. Coaches, not only in professional football but also in youth development, should pay particular attention during training to the development of technical aspects that have been shown to be more demanding at higher levels of competition, with the aim of preparing players for elite football.

Third, greater physical performance, especially in high-intensity actions, is also necessary for certain positions in higher divisions. Therefore, practitioners may need to adopt a position-specific approach to player conditioning within their teams to support the coach’s tactical model and to minimise injury risk by delaying the onset of fatigue.

Further investigations could focus on players transitioning from the Second to the First Division to examine whether their physical and technical adaptations align with this study’s positional trends. Recent research has shown significant shifts in high-intensity running and technical involvement over multiple seasons [43]. In addition, future studies could employ multivariate analyses integrating physical, technical, and contextual variables, which may help to better understand differences between players’ competitive standards. Moreover, the role of explosive actions (e.g., accelerations and decelerations) should be explored, as they may represent critical success factors at higher competitive levels. Finally, it would be valuable to examine how possession styles influence physical and technical profiles and how they interact with tactical and contextual factors to better understand performance determinants in elite football.

## CONCLUSIONS

The study results demonstrate that players’ physical and technical demands are influenced by their competitive standard. Specific playing positions required different high-intensity and sprint demands for WD, CM, and FW across the different phases of play, with ball possession being an important factor influencing running demands. Central defenders and WM did not show differences in physical demands between divisions. However, different technical requirements were observed across all positions. In general, First Division players, particularly FW and WM, were distinguished by superior offensive technical performance, such as passes and successful attacking actions. The findings of this study indicate that greater technical demands during the attacking phase across all positions, as well as high-intensity and sprint-related physical aspects in WD, CM, and FW, may explain the differences between players at higher and lower competitive standards.
